# Insights Into the Role of Endoplasmic Reticulum Stress in Infectious Diseases

**DOI:** 10.3389/fimmu.2019.03147

**Published:** 2020-01-31

**Authors:** Ji-Ae Choi, Chang-Hwa Song

**Affiliations:** ^1^Department of Medical Science, College of Medicine, Chungnam National University, Daejeon, South Korea; ^2^Department of Microbiology, College of Medicine, Chungnam National University, Daejeon, South Korea; ^3^Research Institute for Medical Sciences, College of Medicine, Chungnam National University, Daejeon, South Korea

**Keywords:** ER stress, infection, infectious disease, UPR (unfolded protein response), bacteria, viruses, pathogen, apoptosis

## Abstract

The endoplasmic reticulum (ER) is the major organelle in the cell for protein folding and plays an important role in cellular functions. The unfolded protein response (UPR) is activated in response to misfolded or unfolded protein accumulation in the ER. However, the UPR successfully alleviates the ER stress. If UPR fails to restore ER homeostasis, apoptosis is induced. ER stress plays an important role in innate immune signaling in response to microorganisms. Dysregulation of UPR signaling contributes to the pathogenesis of a variety of infectious diseases. In this review, we summarize the contribution of ER stress to the innate immune response to invading microorganisms and its role in the pathogenesis of infectious diseases.

## Introduction

The endoplasmic reticulum (ER) is crucial for maintaining cellular calcium homeostasis and for the production, processing, and transport of proteins and lipids (1). The rough ER working with membrane-bound ribosomes produces the protein and continues protein assembly, while the smooth ER synthesizes lipids, phospholipids, and steroids (2). The ER is sensitive to stresses that perturb the intracellular energy level, redox state, or calcium concentration. If the protein-folding function of the ER is reduced by stresses, unfolded proteins or misfolded proteins can accumulate. To prevent the resulting cytotoxicity, the unfolded protein response (UPR) is activated (1). ER-resident transmembrane proteins such as inositol-requiring enzyme 1 (IRE1), protein kinase R (PKR)-like ER kinase (PERK), and activating transcription factor 6 (ATF6) are implicated in activation of the UPR (3).

To maintain protein-folding homeostasis in the ER, mRNA translation is transiently attenuated through phosphorylation of eIF2α leading to ATF4 activation (2). IRE1α-dependent decay (RIDD) suppresses the load of new synthesized protein in ER through degradation of ER-localized mRNAs, ribosomal RNAs, and miRNAs (4, 5). Spliced X-box binding protein-1 (XBP-1) mRNA induced by activated IRE1α regulates the expression of numerous target genes including ER chaperones and ER-associated protein degradation (ERAD) components (6, 7). For instance, BiP is known to be an important factor in modulating the UPR to avoid apoptosis (4). ER stress regulates autophagy by modulating the release of calcium from the ER to cytosol and by modulating the activation of mTORC via PI3K/AKT or AMPK (8). Autophagy is also essential to maintain cellular homeostasis through degradation of dysfunctional components from the cells by using lysosomal degradation pathway (8, 9). The activation of three main molecules of UPR induces autophagy via regulation of ATG genes (8). The spliced XBP-1 directly binds to beclin-1 gene promotor region (10).

Another clearance mechanism to degrade the accumulation of misfolded proteins is the ERAD pathway. ERAD substrates recognized by ER chaperone are delivered to ERAD adaptors on ER membrane and then it is retro-translocated into the cytosol (11). The misfolded proteins undergo proteolytic degradation by the ubiquitin proteasome system to maintain the proteostasis (11). When the accumulation of misfolded proteins in the ER overwhelms the capacity of the ERAD system, ER stress, detected by the ER stress sensors IRE1, ATF6, and PERK, activates the UPR, but excessive ER stress may eventually lead to apoptosis (1, 12). Although the mechanism of UPR is well-known, it is not clear how the response regulates both apoptotic and adaptive pathways. A previous report suggested that instabilities of pro-survival and pro-apoptotic mRNAs and proteins mediate adaptation to ER stress (13). It was proposed that Death Receptor 5 (DR5) integrates dynamic UPR signals to control apoptosis in relation to ER stress (14).

In stressed cells, unfolded and misfolded proteins may accumulate in ER and ER-folding capacity is exceeded, causing apoptosis (15). PERK and eIF2α phosphorylation play an important role in protecting cells against the consequences of ER stress (16). Diverse stress stimuli-induced eIF2α dephosphorylation causes cells to die due to ER stress (12). Although C/EBP Homologous Protein (CHOP) is a well-known transcription factor induced by eIF2α phosphorylation, deregulated CHOP expression promotes apoptosis (17). CHOP promotes the expression of Bim, a pro-apoptotic protein, and decreases the expression of anti-apoptotic Bcl-2 (18). The GADD34, transcriptional target of CHOP, induces eIF2α dephosphorylation leading to restoration of protein translation (12). However, the overexpression of GADD34 can elicit apoptosis (12). Hyperactivated IRE1α cleaves and degrades precursor of miRNAs that normally repress translation of caspase-2 mRNA, and thus induces mitochondrial apoptotic pathway (19). Disruption of the cellular Ca^2+^ homeostasis induces calpain activation, which cleaves Bid and pro-caspase-12, and subsequently triggers caspase-3-dependent apoptosis (20).

It is well-known that ER stress is associated with the pathogenesis of various diseases such as obesity, diabetes, cancer, neurodegenerative disorders, inflammatory diseases, and infectious diseases. However, we have only vague ideas of how ER stress is involved in the pathogenesis of infectious diseases. Recently, many scientists are trying to unveil the implication of ER stress in infectious diseases. Thus, a better understanding of the regulatory mechanisms of ER stress will be important in the development of new therapeutics to treat refractory infectious diseases.

## The UPR in Immunity

ER stress induces an inflammatory response by activating UPR transcription factors and plays an important role in the pathogenesis of inflammatory and autoimmune diseases, such as obesity, diabetes, atherosclerosis, myositis, and inflammatory bowel disease (21). The UPR has important roles in the development of immune cells because UPR regulates immune cell differentiation, activation, and cytokine production (21). The immunostimulant lipopolysaccharide (LPS) induces inflammatory cytokine production and activates the transcription of ER chaperone genes, including spliced XBP-1, BiP, ATF4, and CHOP (21, 22). Spliced XBP1s in response to toll-like receptor (TLR) is necessary for macrophages to produce proinflammatory cytokines (23). It has been known that the IRE1α-XBP-1 pathway activates production of TNF and IL-6 in macrophages of cystic fibrosis patients (24). Additionally, the IRE1α/TRAF2 pathway-mediated NOD1 and NOD2 signaling provides ER-stress-induced inflammation (25). Many studies have shown that the role of ER stress is associated with immune cell differentiation, activation, and cytokine production.

A recent report suggests that XBP-1 is important for the differentiation of Th17 cells (26). Interestingly, it is known that the IRE1–XBP-1 pathway is activated by acute infection and is required for T cell differentiation (27). Another role of the IRE1–XBP-1 pathway is to support the functions of plasma cells and the survival of dendritic cells (28, 29). UPR is implicated in host immunity due to its involvement in calcium signaling, glycosylation, lipid metabolism, and oxidative protein folding (30). Activation of the T-cell receptor, the B-cell receptor, the Fc-γ receptor, and various cytokine receptors causes calcium efflux from the ER through inositol 1,4,5-trisphosphate receptor (IP3R) (30). The resulting increased intracellular calcium concentration activates cellular signaling molecules to promote T-cell activation, maturation of myeloid cells, and cellular differentiation, adhesion, and death (31). The increased levels of ROS triggered by ER stress activate not only proinflammatory signals but also inflammasome formation, suggesting that ER stress exerts immunogenic effects (32).

The transcription factor nuclear factor kappaB (NF-κB) regulates the immune response of the host. ER-stress-mediated activation of NF-κB may modulate the production of cytokines (21). The IRE1α-TRAF2–IKK interaction is known to activate NF-κB, which in turn leads to the production of TNF-α (33). IRE1α induces inflammation by activating JNK (34, 35). The interaction between ER stress and MAPKs (JNK, p38, and ERK) may contribute to inflammatory responses. Therefore, the UPR plays a critical role in regulating the immune response.

## Relationship of ER Stress With Pathogens

In mammalian cells, the UPR is triggered by three ER-stress sensor proteins, IRE1, PERK, and ATF6, to restore ER homeostasis (3). Infection by the majority of known pathogens activates the UPR. Modulation of the functions of the ER by pathogens can result in their survival/replication or clearance because ER stress is associated with autophagy or apoptosis. Although little is known about the role of the ER-stress response in the pathogenesis of viral and bacterial infection, the regulation of ER stress might be important in intractable infectious diseases.

### Bacterial Infection and ER Stress

**Figure 1:**
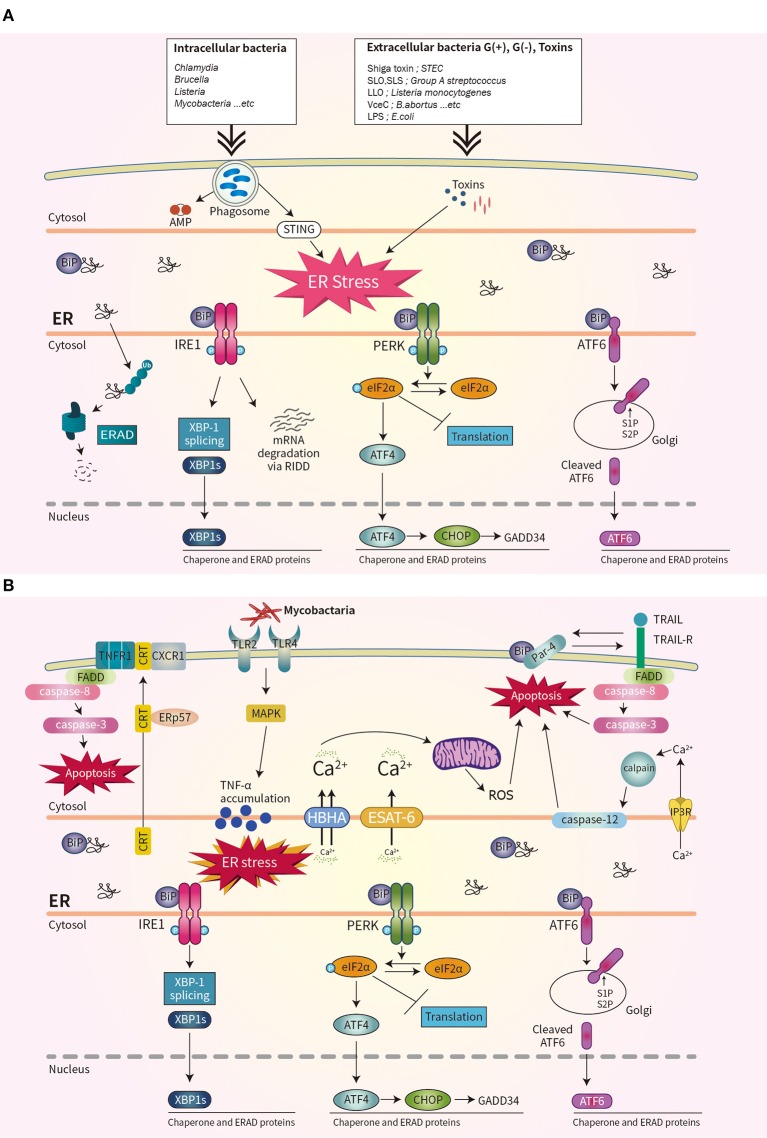
Schematic overview of unfolded protein response (UPR) signaling during bacterial infection. **(A)** Three ER stress sensors–IRE1, PERK, and ATF6–are activated when the accumulation of misfolded protein aggregates promotes recruitment of BiP. Bacterial infection and toxins activate the UPR. **(B)** During mycobacterial infection, co-translocated calreticulin and ERp57 form a complex with TNFR1 and CXCR1 in the plasma membrane, leading to apoptosis, and suppression of intracellular Mtb. The interaction of Par-4 and BiP leads to apoptosis by inducing Mtb-mediated ER stress and activating the FADD/caspase-8/-3 pathway. The mycobacterial antigens HBHA and ESAT-6 affect the ER membrane and induce the release of Ca^2+^ from the ER to mitochondria, leading to the production of reactive oxygen species and apoptosis.

Bacterial virulence factors are involved in UPR activation. LPS from Gram-negative bacteria binds to TLR4 that is delivered by Glucose-regulated protein 94 (Grp94) (36). The expression of Grp94 (an HSP90-like protein specialized for protein folding and quality control in the ER) is increased by LPS stimulation (36). The cytotoxin subtilase, produced by Shiga-toxigenic *Escherichia coli*, cleaves BiP, resulting in DNA fragmentation and UPR-mediated apoptosis (37). Similarly, the Shiga toxin produced by the enteric pathogens *Shigella dysenteriae* serotype 1 and enterohemorrhagic *E. coli* increases ER-stress-mediated apoptosis by inducing release of Ca^2+^ from the ER to the cytosol and upregulating PERK-CHOP-mediated DR5 (38, 39). Subunit A of unfolded cholera toxin (CT) retro-translocates through the ER membrane to the cytoplasm, where it directly binds to the ER-luminal domain of IRE1α (40). Streptolysin O and streptolysin S of group A *Streptococcus* (GAS) induce the production of ATF4, which upregulates the expression of proliferation-related genes of GAS (41). ER stress is also important for biofilm formation, microcolony aggregation, distribution, and spread of GAS during infection of soft tissues (42). The pore-forming toxin listeriolysin O (LLO) produced by *Listeria monocytogenes* induces the three axes of the UPR before cell entry (43). Therefore, diverse bacterial taxa induce the UPR by secreting toxins (Figure 1A).

The obligate intracellular pathogen *Chlamydia* induces the UPR by upregulating BiP (44, 45). *Chlamydia* infection also induces TLR4/IRE1-mediated activation of PKR, which enhances IFN-β production (46). *Brucella abortus* localizes to the ER by transforming its fine reticular pattern into a thicker tubular structure (47). Also, VceC of *B. abortus* directly binds BiP and selectively activates the IRE1–XBP-1 pathway, which increases the IL-6 level (47). Enhancement of the UPR by co-stimulation with IFN-β promotes the replication of *B. abortus* in host cells (48). Thus, modulating the UPR may be useful for treating brucellosis or chlamydia infection (Figure 1A).

Bacterial infections are frequently caused in chronic diseases such as obesity, type 1 and type 2 diabetes (T2D), and atherosclerosis (49, 50). The reason that altered immune functions in these chronic diseases are observed is because the immune system can be affected by chronic stress (15, 50). It has been known that different ER stress sensors are activated during bacterial infection (11). Therefore, understanding the diverse roles of ER stress sensors during bacterial infection might be effective to treat chronic diseases in the future.

### Mycobacterial Infection and ER Stress

*Mycobacterium tuberculosis* (Mtb) is the causative agent of tuberculosis (TB), does not produce toxins, and grows very slowly. However, Mtb infection induces ER stress in host cells. Mycobacterial ESAT-6 and HBHA induce ER stress by promoting ROS production, by disrupting intracellular calcium homeostasis (51, 52). ER-stress-induced apoptosis suppresses the intracellular growth of Mtb by activating caspase-12 in the outer membrane of the ER (52). Phosphorylation of eIF2α is reported to be an important component of the ER stress response that modulates the intracellular survival of Mtb (Figure 1B) (53).

Interestingly, Mtb-induced ER chaperones contribute to the translocation of CRT or Par-4 to the plasma membrane of macrophages, leading to apoptosis and suppression of Mtb growth (54, 55). Also, mycobacterial infection induces an ER-stress response due to accumulation of misfolded or unfolded TNF-α in the ER; this indicates that Mtb-mediated overproduction of proinflammatory cytokines induces ER stress in macrophages (Figure 1B) (56). Moreover, activation of the RIDD pathway suppresses the intracellular growth of mycobacteria (57). Therefore, investigation of the regulatory mechanism of ER stress during mycobacterial infection might suggest new therapeutic targets for multidrug-resistant TB.

### Viral Infection and ER Stress

Viruses modulate host defense mechanisms to escape the host immune response. Viruses may interact with the host UPR to maintain an environment favorable for establishment of persistent infection. Indeed, viral infection can disturb ER stress (58). ER stress and the UPR are reported not to protect against infection by reovirus and hepatitis B virus but, rather, promote their replication (59, 60).

**Figure 2:**
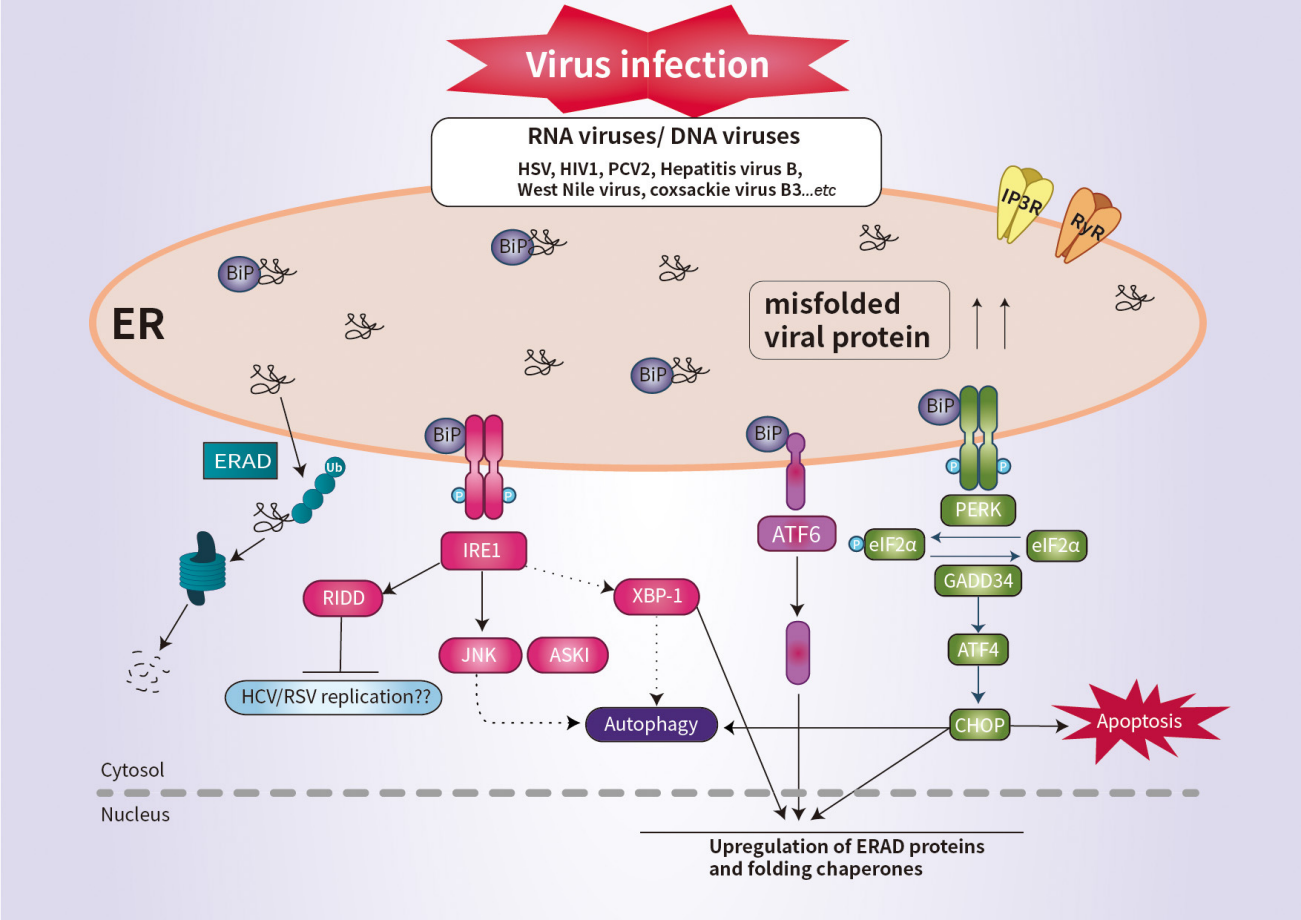
Schematic overview of UPR signaling during viral infection. Viral infection induces ER stress and the UPR, which promotes cell survival by inhibiting apoptosis. Some viral infection induces ER-stress-mediated apoptosis by promoting the synthesis of CHOP. IRE1 activates RIDD to promote the degradation of ER-localized mRNAs. The activation of RIDD may enhance viral protein synthesis. The interaction between the UPR pathways and the autophagic response is implicated in the pathogenesis of viral infection.

The PERK pathway is important for host antiviral defense (61). The PERK-mediated phosphorylation of eIF2α may be responsible for regulating viral replication (62). Similarly, regulation of the phosphorylation of eIF2α is important for the survival of enveloped viruses, such as herpes simplex virus (HSV) (63). HSV reduces the level of MHC-I by promoting its ER-associated degradation (ERAD), leading to suppression of the immune response (64). Infection by human immunodeficiency virus type 1 induces the degradation of CD4 by ERAD (64). RIDD degrades mRNAs to reduce ER load and alleviates ER stress (9). Although it is unclear whether RIDD activation is beneficial for all viral infectivity, RIDD is closely associated with viral RNA synthesis (4, 9). This is likely because virus replication requires large quantities of membrane proteins and lipids, which are produced in the ER (Figure 2).

CHOP plays an important role in suppressing infection of host cells by RNA viruses (9). For example, porcine circovirus type 2 (PCV2) triggers the eIF2α-ATF4–CHOP pathway and activation of caspases (65). West Nile virus and coxsackie virus B3 induce ER-stress-mediated apoptosis by promoting the synthesis of CHOP (66, 67). Interestingly, IRE1 is reportedly essential for induction of autophagy during infection with infectious bronchitis virus (Figure 2) (68). Thus, further investigation of the ER stress response would enhance our understanding of the pathogenesis of viral infection.

## Conclusion and Future Perspectives

Knowledge of the mechanisms by which viruses modulate the UPR has advanced more than that of bacteria. The interaction between pathogenic bacteria and ER stress is under active investigation, but the role of ER stress in the pathogenesis of infectious diseases is unclear. How ER stress modulates bacterial survival and how bacteria modulate ER stress to promote their replication need to be studied. ER stress is not only associated with autophagy but also with the immune response to pathogens. Targeting ER stress and the UPR with small molecules is emerging as a promising therapy for treatment of various diseases such as neurodegeneration, cancer, metabolic diseases, stroke, and heart disease (69). Therefore, studies of the regulatory mechanisms of ER stress during pathogenic infection are warranted. The results of such efforts are likely to lead to the development of novel host-derived therapeutics for infection by multidrug-resistant bacteria or emerging viruses.

## Author Contributions

J-AC and C-HS contributed to the writing of the manuscript, proofreading, editing, and figure preparation.

### Conflict of Interest

The authors declare that the research was conducted in the absence of any commercial or financial relationships that could be construed as a potential conflict of interest.
